# Effects of Resveratrol on the Structure and Catalytic Function of Bovine Liver catalase (BLC): Spectroscopic and Theoretical Studies

**DOI:** 10.15171/apb.2017.042

**Published:** 2017-09-25

**Authors:** Samaneh Rashtbari, Gholamreza Dehghan, Reza Yekta, Abolghasem Jouyban, Mehrdad Iranshahi

**Affiliations:** ^1^Department of Biology, Faculty of Natural Sciences, University of Tabriz, Tabriz, Iran.; ^2^Pharmaceutical Analysis Research Center and Faculty of Pharmacy, Tabriz University of Medical Sciences, Tabriz, Iran.; ^3^Department of Pharmacognosy, Faculty of Pharmacy, Mashhad University of Medical Sciences, Mashhad, Iran.

**Keywords:** Bovine liver catalase, Molecular docking, Spectroscopy, Trans resveratrol, Uncompetitive inhibition

## Abstract

***Purpose:*** The study on the interaction between various compounds and macromolecules such as enzymes has been very important for monitoring the alteration of structural and functional properties of them. Resveratrol (3, 5, 4-trihydroxy-stilbene; RES) is a biologically active phytoallexin found in grapes and other food products. This article shows an interaction of native bovine liver catalase (BLC) with natural antioxidant product, trans resveratrol (tRES) using multispectroscopic methods.

***Methods:*** The interaction between BLC and tRES is performed using UV-vis absorption, fluorescence and circular dichroism (CD) spectroscopy and molecular docking study.

***Results:*** In vitro kinetic studies indicated that tRES can decrease BLC activity through uncompetitive inhibition. The results of spectroscopic methods represented that the binding of tRES with BLC can change the micro-region around aromatic amino acids (tryptophan (Trp) and tyrosine (Tyr)) and quench intrinsic fluorescence of BLC by a static mechanism. According to fluorescence quenching data analysis, it was revealed that tRES has one binding site on BLC. The thermodynamic parameters were obtained, which demonstrated that tRES can bind to BLC by van der Waals forces and hydrogen bonds. Molecular docking results indicated that tRES binds to BLC away from heme group and near to the Tyr 324 and Phe 265. These results are in agreement with the experimental results.

***Conclusion:*** The inhibitory effect of tRES on BLC demonstrated that excessive consumption of the antioxidants could be resulted in hazardous effects.

## Introduction

Catalase (H_2_O_2_:H_2_O_2_ oxidoreductase, EC 1.11.1.6), one of the main components of the antioxidative defense system, is an active and ubiquitous enzyme that exists in almost all living organisms. It protects human and animal tissues against toxic effects of hydrogen peroxide (H_2_O_2_) that acts as a free radical.^1,2^ Catalase deficiency leads to many disorders such as aging, mutagenesis, acatalasemia, urinary tract diseases, diabetes, Alzheimer’s disease, vitiligo and tumors.^3^

Bovine liver catalase (BLC) is a homotetramer enzyme with four subunits. Each subunit has over 506 amino acids, one NADPH molecule (as a cofactor) and protoporphyrin IX (the active-site heme group) containing a Fe^3+^.^4,5^ Heme group is vital for enzymatic reactions. Also, each subunit has four domains: β-barrel, N-terminal threading arm, wrapping loop and C-terminal helices. The β-barrel core structures are conserved in all catalases and heme group with a histidine (His), a tyrosine (Tyr) and an asparagine (Asn) is buried in the core structure.^4,6^ Catalase binds NADPH at a cleft between the β-barrel and helical domain on the surface of the molecule. NADPH is not necessary for the activity of catalase and prevents inactivating of enzyme at low concentration of H_2_O_2_.^4^

There are three channels in catalase structure, which one of them reaches the active site. Through this channel substrate (H_2_O_2_) can enter and products can exist. Catalase catalyzes dismutation of H_2_O_2_ in two-step: in the first step, equation (1), one H_2_O_2_ molecule is reduced by ferricatalase (resting form of the enzyme), producing oxy-ferryl intermediate with a porphyrin π-radical cation, compound I, and H_2_O molecule. In the second step, equation (2), compound I is reduced by another H_2_O_2_ molecule, result in formation resting form of enzyme and generation of O_2_ and second H_2_O molecule.^7-9^

(1) $$E_{}(por-Fe^{III})+H_{2}O_{2}\to Compound_{}I_{}(por^{•+}-Fe^{IV}=O)+H_{2}O$$

(2) $$Compound_{}I_{}(por^{•+}-Fe^{IV}=O)+H_{2}O_{2}\to E_{}(por-Fe^{III})+H_{2}O+O_{2}$$

Human erythrocyte catalase (HEC) is very similar (similarity of 91%) to BLC, with the only difference in 43 amino-acid residues at the C-terminus domain.

**Figure 1:**
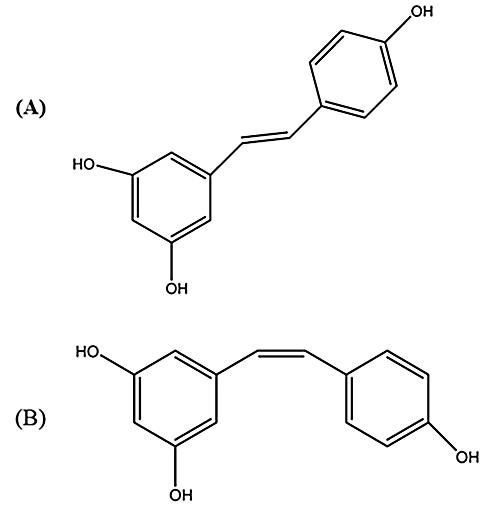
Resveratrol (3, 5, 4-trihydroxy- stilbene); (A) *trans*-(E) resveratrol, (B) *cis*-(Z) resveratrol.

Resveratrol (3,5,4-trihydroxy-stilbene; RES), as a natural polyphenolic product, belong to a group of phytochemicals called "stilbenoid".^10^ Stilbenoids are a subclass of phytoalexins and are produced by plants in response to injury, insect infestation, pathogens attack and ultraviolet exposure. RES found as two configurations: *trans*-(E) and *cis*-(Z)^11^ (Figure 1). The *trans* form is more active and stable than *cis* form. It widely exists in most of the foods, particularly in grape skin, cranberries, blueberries and red wine.^12^ *In vivo* studies showed that RES has beneficial effects such as anticancer effects that inhibit growth and proliferation of cancer cells by inhibiting angiogenesis and inducing apoptosis.^13^ It has other beneficial effects on the human body, including antioxidant activity (in the treatment of diabetes and obesity),^14^ cytoprotective,^15^ anti-inflammatory^16^ and anti-aging effects. Also, recently RES has shown neurodegenerative effects in experimental animals against neurological disorders such as Huntington, Parkinson and Alzheimer diseases.^17^ Shen et al. reported that RES can inhibit α-amylase and α-glucosidase in an uncompetitive manner.^18^

The aim of this work was to investigate the possible effects of tRES on the structure and catalytic function of catalase by different methods. Since catalase exists at a high level in liver and this organ is one of the most important organs in the body, in which are metabolized and detoxified chemical and natural compounds, therefore study the side effects of varies drugs and compounds on catalase can be important. In this work we focus on the effects of tRES, as a natural occurring stilbene in fruits and beverages, on the structure and the activity of BLC.

## Materials and Methods

Bovine liver catalase (BLC,‏ MW; 250 KD) and dimethylsulfoxide (DMSO) were obtained from Sigma-Aldarich (St. Louis, MO, USA). Hydrogen peroxide (H_2_O_2_) 30%, disodium hydrogen phosphate (MW; 177.99 g.mol^-1^) and sodium dihydrogen phosphate (MW; 156.02 g.mol^-1^) used in the buffer preparation were obtained from Merck Co. (Darmstadt, Germany). *Trans* resveratrol was purchased from Changsha Herbal Ingredient Co. (Changsha, China). The tRES stoke solution (8.7 mM) was prepared by dissolving in DMSO. The concentration of the H_2_O_2_ stock solution was calculated by using its absorbance at 240 nm and the extinction coefficient of 40 M^-1^ cm^-1^.^19^ All chemicals used for the assays were chemically pure.

### Kinetics studies of the native BLC

BLC activity was measured spectrophotometrically (T60, PG Instruments LTD., Leicestershire, UK) using the decrease in H_2_O_2_ maximum absorbance‏ at 240 nm (A_240_) due to its degradation by BLC.^20^ In order to measurer the BLC activity, the reaction mixture containing 50 mM sodium phosphate buffer (pH 7) and an appropriate amount of H_2_O_2_ (10-90 mM) were added to three ml cuvette. The enzymatic reactions were started by adding 10 μl of BLC suspension (3 nM) to the mixtures. Changes at A_240_ were recorded every two second for one minute and these alterations were considered as BLC activity.

In order to determine the possible effects of tRES on BLC activity, 3 nM of BLC in sodium phosphate buffer (pH 7) was incubated with various concentrations of tRES (2.9, 5.8, 8.7, 11.6 and 14.5 μM ). Subsequently, fixed concentration of H_2_O_2_ (60 mM) was added to the incubated solutions and changes in A_240_ were recorded.

### Spectroscopic studies

The UV-vis absorption spectra of BLC were recorded by UV-visible spectrophotometer (T60, PG Instruments LTD., Leicestershire, UK) in the range of 200-500 nm. In order to investigate the effects of tRES on BLC structure, its absorption spectra were recorded in the presence of different concentrations of tRES.

In order to monitor the structural changes of BLC in the presence of different concentrations of tRES (0.65, 1.31, 1.97, 2.62, 3.28, 3.94 and 4.59 μM), all fluorescence spectra were recorded on (Jasco, FP-750 spectrofluorometer, Kyoto, Japan) at two temperatures (25 °C and 37 °C). The excitation wavelength (λ_ex_) was fixed at 295 nm and emission wavelength (λ_em_) was recorded from 300-500 nm.

According to the inner filter effect (IFE) definition, if a sample shows significant absorption at excitation and emission wavelengths, these effects should be corrected. Since IFE can be neglected for weak absorbance (i.e. weak concentrations), a common procedure is to dilute the solution until maximal absorbance is inferior than 0.1. Also, when the absorbance of the solution is lower than 0.3 the equation (3) can be used to correct the IFE.^21^

(3) $$F_{cor}=F_{obs}\times 10^{\frac{\left(A_{ex}+A_{em}\right)}{2}}$$

where *F*_cor_ and *F*_obs_ are the correct and observed fluorescence intensity, respectively. *A*_ex_ and *A*_em_ indicate the sample (tRES) absorbance at the excitation and emission wavelength, respectively.

In the present work, we diluted the stock solution of tRES until maximal absorbance at excitation wavelength (295 nm) and maximum emission wavelength of BLC (332 nm) was lower than 0.3.

Synchronous fluorescence spectra of BLC in the presence or absence of different concentrations of tRES was recoded using Δλ=15 nm with λ_em_=240-400 for tyrosine (Tyr) and Δλ=60 nm with λ_em_=300-500 for tryptophan (Trp). The scan speed was set at 1000 nm.min^-1^.

Circular dichroism (CD) spectra of BLC in the presence and absence of tRES were measured by a circular dichroism spectropolarimeter (Jasco (J-810), Tokyo, Japan) at 25 °C in 50 mM sodium phosphate buffer, pH 7. In order to estimate the changes in the percentage of secondary structures of BLC under the effects of various concentrations of tRES, the CDNN software was used.

### Molecular docking study

In order to predict the mode of interaction between tRES and BLC, molecular docking calculations were carried out using Auto Dock 4.2 software.^22^ The crystal structure of BLC was obtained from the Protein Data Bank (PDB Id: 1TGU). The molecular structure of tRES was generated using Hyperchem 8.0.6 program and was optimized for minimal energy by Gaussian 98 program. The Lamarckian genetic algorithm was applied to searching the optimum binding site of tRES to the BLC.^23^ In order to recognize the binding sites in BLC, blind docking was carried out with setting of grid box size 126 A˚×126 A˚×126 A˚ grid points and 0.375 A˚ spacing. The VMD and Auto Dock Tools 1.5.4 packages were used for theoretical analysis of modes of interaction between BLC and tRES.^24^

## Results and Discussion

### Kinetics studies

#### Analysis of BLC activity

BLC breakdowns H_2_O_2_ to H_2_O and O_2_. Decomposition of H_2_O_2_ by BLC‏ leads to decrease in H_2_O_2_ UV absorption and this reduction was‏ considered as BLC activity. For this purpose, a fixed concentration of BLC (3 nM) in the presence of additional concentration of H_2_O_2_ was used. The results showed that with increasing H_2_O_2_ concentration, enzyme activity increases (at low H_2_O_2_ concentration) and a linear relationship between enzyme activity and substrate concentration was observed (from 10 to 70 mM) but at higher concentrations of H_2_O_2_ (more than 70 mM), a marked reduction in enzyme activity was observed. This reduction is due to suicide inactivation of BLC by H_2_O_2_.^20^ Michaelis-Menten and Lineweaver-Burk graphs were plotted and kinetic parameters were calculated according to Lineweaver-Burk graph and equation (4):

(4) $$\frac{1}{V_{}}=\frac{k_{m}}{V_{\max}}.\frac{1}{\left[S\right]}+\frac{1}{V_{\max}}$$

The *V*_max_ and *K*_m_ values were determined to be 2.28 mM.S^-1^ and 39 mM, respectively. Using the equation (5) the value of the catalytic constant (*K*_cat_) was obtained as 7.6 × 10^5^ S^-1^.

(5) $$V_{\max}=k_{cat}.[E_{t}]$$

#### Effect of tRES on BLC activity

**Figure 2:**
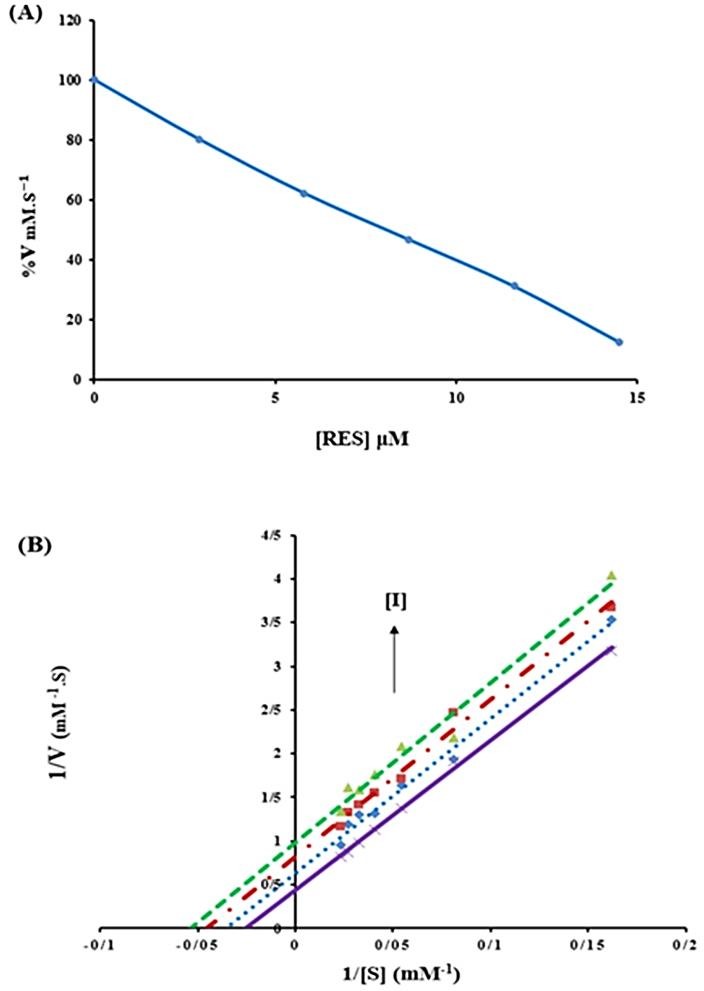
(A) The effect of various concentrations of tRES (0, 2.9, 5.8, 8.7, 11.6 and 14.5 µM) on the activity of BLC in 50 mM phosphate buffer, pH 7 at 25 °C. (B) Lineweaver-Burk plot of BLC (3 nm) with and without various concentrations of tRES: 0 (×), 4.05 (▪), 8.1 (■) and 16.2 (▲) µM in 50 mM phosphate buffer, pH 7, 25 °C.

In order to monitor the effect of tRES on BLC activity, enzyme activity was determined in the presence of various concentrations of tRES (2.9, 5.8, 8.7, 11.6 and 14.5 μM) and fixed concentration of H_2_O_2_ (70 μM). We observed that with increasing concentration of tRES, BLC activity significantly decreased (Figure 2A). The IC_50_ value (the concentration of an inhibitor that inhibits 50% of the enzyme activity) was calculated as 8.1 μM (Figure 2A).

There are three types of reversible enzyme inhibition mechanism: competitive, in which inhibitor binds to the free enzyme and competes with the substrate, non-competitive in which inhibitor interacts with both the free enzyme and enzyme-substrate (ES) complex and uncompetitive (also named anticompetitive inhibition) in which inhibitor only binds to the ES complex.^25^ In order to estimate the enzyme inhibition mechanism, enzyme activity in the presence of different concentrations of tRES was measured and Lineweaver-Burk graphs were plotted. According to these plots and using equation (6), the kinetic parameters (*K_m_^app^* and *V_m_^app^*) were calculated (Table 1) and compared with kinetic parameters of untreated BLC (Table 1). Lineweaver–Burk equation for uncompetitive inhibition is:^26^

(6) $$\frac{1}{V_{0}}=\frac{k_{m}}{V_{\max}}.\frac{1}{\left[S\right]}+\frac{1}{V_{\max}}.(1+\frac{\left[I\right]}{K_{i}})$$

The results showed that with increasing concentration of tRES,*K_m_^app^* and *V_m_^app^* values decreased, which is characteristic of an uncompetitive inhibitor (Figure 2B).

**Table 1:** The V_m_ and K_m_ values of BLC with and without tRES.

**Concentration of RES(µM)**	**V**_m_ **(mM.s**^-1^)	**K**_m_ **(mM)**
0	2.28	39
4	1.57	27.7
8	1.21	21.69
16	1.01	18.48

### UV-vis absorption spectrophotometry

**Figure 3:**
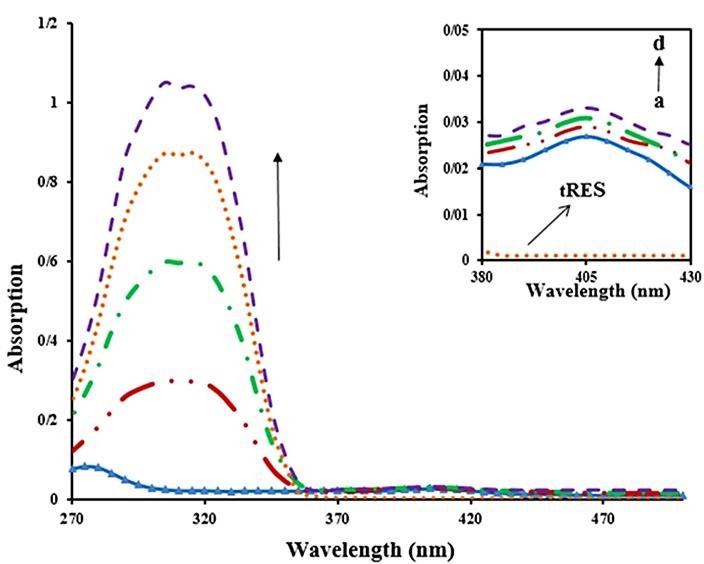
UV-vis absorption spectra of BLC (1μM) in the absence and presence of additional concentration of tRES and a magnified image of the Soret-bands: (a) 0, (b) 4.05, (c) 8.1 and (d) 16.2 µM in 50 mM phosphate buffer, pH 7, 25°C.

UV-vis spectrophotometry, as an impressive and simple technique, is used to monitoring the conformational changes of macromolecules (such as proteins) that occur upon interaction with various ligands.^27^ Most of the proteins such as BLC show main absorption bands around 280 nm, which are caused by the existence of aromatic amino acids including tryptophan (Trp), phenylalanine (Phe) and tyrosine (Tyr).^28^ Also, BLC has an important absorption peak around 405 nm (Soret-band) due to π→π^*^ transition of electrons in the porphyrin ring of heme group. In this study, UV-vis absorption spectra of BLC were recorded (1 μM) in the presence of three concentrations of tRES (4.05, 8.1 and 16.2 μM). Figure 3 shows that in the presence of additional concentration of tRES, absorption intensity around 405 nm (Soret-band) increases regularly (a little hyperchromic shift) compared with the native enzyme. Slight increase in the absorption peak of Soret-band indicates that no significant structural change of BLC occurs and tRES cannot directly bind to the deeply buried heme group. The Soret-band is sensitive to alterations of the microenvironment of heme group, so a small displacement of Soret-peak observed in the presence of tRES, indicating a little alteration in the microenvironment of tryptophan residue.^29,30^ Due to the overlapping of BLC and tRES bands around 280 nm, it was not feasible to estimate the alteration at 280 nm, therefore we used fluorescence spectroscopy.

### Fluorescence spectroscopy

This method is used to conformational changes studies of proteins. Three aromatic amino-acids (Trp, Tyr and Phe) exist in protein structure and contribute to their intrinsic fluorescence emission. The emission of tryptophan is highly dependent on polarity and local environment whereas the emission of tyrosine is insensitive to solvent polarity. The effect of Phe on protein’s fluorescence is less than others.^28^ In order to investigate the effect of tRES on fluorescence intensity of BLC (0.7 μM), the additional concentration of tRES (0-5.0 µM) was used and excitation wavelength was set at 295 nm. Also, the studied emission wavelength was 322 nm (maximum emission wavelength of BLC).

**Figure 4:**
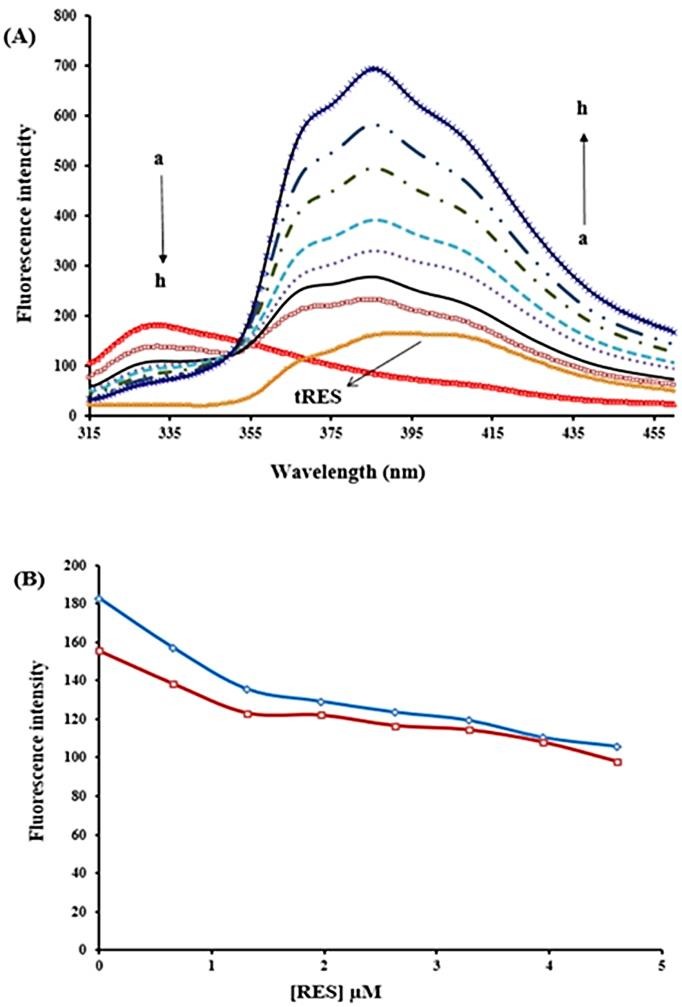
(A) Effect of various concentrations of tRES on fluorescence intensity of BLC. λ_ex_=295, BLC concentration: (0.7 µM); tRES concentrations:(a) 0, (b) 0.65, (c) 1.31, (d) 1.97, (e) 2.62, (f) 3.28, (g) 3.94, and (h) 4.59 µM, (I) only RES, 0.65 µM. (B) fluorescence quenching of BLC in the presence of various concentrations of tRES after correcting inner filter effect, pH 7.4, T=298K (♦), T=310K (■).

When the excitation wavelength is set at 295 nm, the effect of Tyr is overlooked. We observed that tRES leads to quenching of intrinsic fluorescence of BLC due to alteration in the surrounding environment of Trp. Also, the results suggest that tRES and BLC form a new complex (Figure 4A). Since tRES had significant absorption at excitation and maximum emission wavelength of BLC, the inner filter effects were corrected according to equation (3) (Figure 4B).

Quenching mechanisms (static and dynamic quenching) are determined using their different dependence on temperature. In static quenching, the bimolecular quenching constant decreases with increasing temperature. Dynamic fluorescence quenching is a diffusion process and therefore the value of quenching constants increase with increasing temperature.^31^ As shown in (Figure 4B), the quenching mechanism is static. In order to confirm the quenching mechanisms, Stern-Volmer equation (equation (7)) was used:^32^

(7) $$\frac{F_{0}}{F}=1+K_{SV}[Q]=1+K_{q}\tau_{0}[Q]$$

where *F* and *F*_0_ are the fluorescence intensities of BLC with and without the quencher (tRES), respectively, [*Q*] is the concentration of the quencher, *K*_q_ is the quenching rate constant of the biomolecule and* K*_SV_ is the Stern–Volmer quenching constant and τ_0_ is the fluorescence lifetime without quencher, which is 10^-8^ s for BLC.^33^

**Figure 5:**
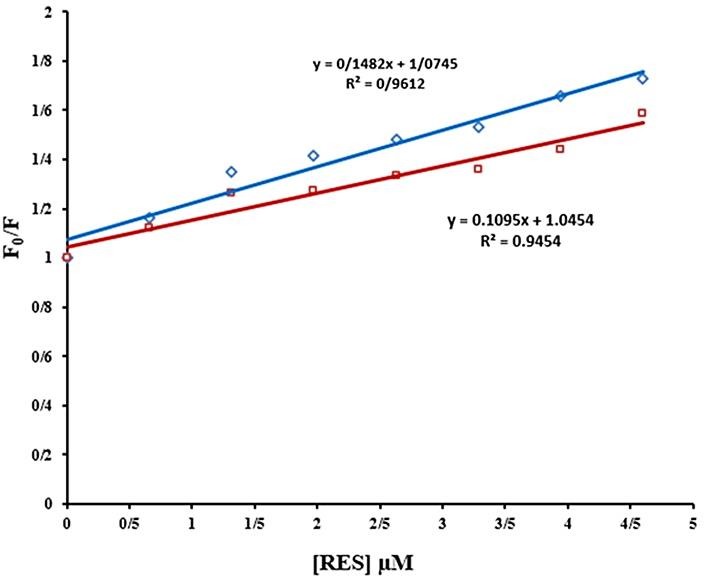
Stern-Volmer curve for quenching of BLC by various concentrations of tRES at 298 K (♦) and 310 K (■).

According to the plot of F0/F *vs*. [*Q*] (Figure 5), the values of *K*_SV_ and *K*_q_ at different temperatures were obtained and summarized in Table 2. The results showed that, the *K*_SV_ values reduce with increasing temperature, and *K*_q_ values are greater than 2×10^10^ L mol^-1^ S^-1^(maximum dynamic quenching constant). This result confirmed that the quenching is mostly static process and fluorescence quenching of BLC is due to formation of BLC-tRES complex in ground state.

**Table 2:** Binding and thermodynamic parameters of BLC and tRES complex at different temperatures (25 °C and 37 °C).

**Temperature (°C)**	**n**	**K**_SV_ ** (×10** ^5^ **M**^-1^)	**K**_q_ ** (×10** ^14^ **M**^-1^**s**^-1^)	**K (×10** ^3^ **M**^-1^)	**ΔG kJmol**^-1^	**ΔHkJmol**^-1^	**ΔS JK**^-1^**mol**^-1^
25	0.71	1.4	1.4	4.9	-21	-40	-63
37	0.69	1	1	2.6	-20		

### Synchronous fluorescence spectroscopy

**Figure 6:**
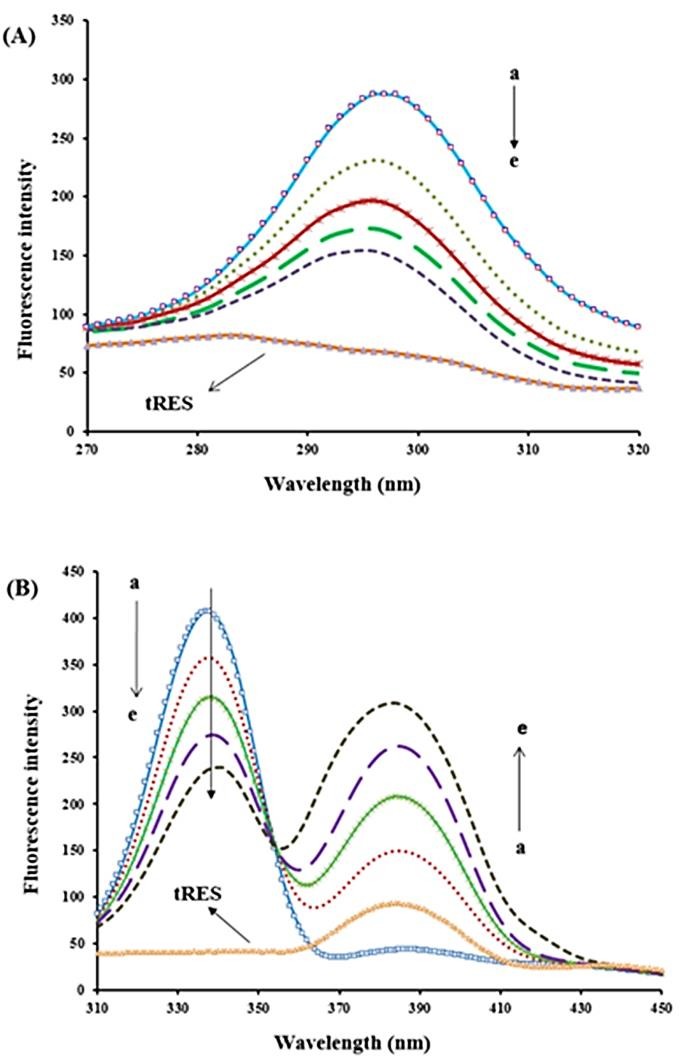
Synchronous fluorescence spectra of BLC in the presence of additional concentration of tRES (a) 0, (b) 0.65, (c) 1.31, (d) 1.97 and (e) 2.62µM; (A) ∆λ=15 and (B) ∆λ=60 nm, (f) only RES 0.6 µM, pH 7.4,T=298K.

Synchronous fluorescence is another simple and sensitive technique that provides important information about the microenvironment in the surrounding of the chromophore and conformational changes in proteins. In this technique, the spectra are recorded by scanning synchronously both emission and excitation wavelength, fixing wavelength interval (∆λ) between them.^34^ The ∆λ can be selected 15 nm or 60 nm and synchronous fluorescence can give main information about microenvironment around the Tyr and Trp, respectively. We observed that synchronous fluorescence spectra of BLC changed under the effect of tRES. As shown in (Figure 6A), with increasing concentration of tRES, the emission spectra of Tyr shifted to the smaller wavelength (blue shift) about 1 nm, which describes a little increase in the hydrophobicity of Tyr, leading to partially expose of heme group to solvent. The emission spectra of Trp shifted to longer wavelength (red shift) about four nm (Figure 6B), demonstrating a decrease of hydrophobicity in Trp micro-region and Trp residue transferred from nonpolar to polar environment.^35^

### Circular dichroism spectroscopy study

**Figure 7:**
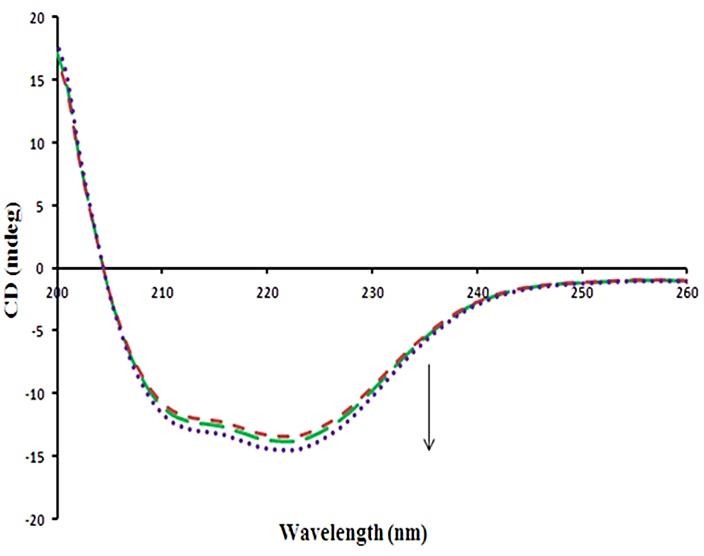
CD spectra of BLC in the absence and presence of tRES at 298K (25 °C).

In order to determine the secondary structure changes of BLC upon tRES binding, CD spectra in the far-UV region were recorded (Figure 7). The CD spectra of BLC show two main negative bands at 208 nm and 222 nm (due to π→π^*^ transition which occurs in the peptide chain). Those are characteristic of an alpha helical structure of the BLC. Also, BLC with β-pleated sheets shows a negative band at 218 nm, while random coils have very low ellipticity above 210 nm.^36^ BLC consist of the secondary conformation of 27.4% α-helix, 21.2% β-plared sheet, 17.6% β-turn and 33.8% random coil. The results showed that secondary structures of BLC change (a little) in the presence of tRES. The effects of tRES on the percentage of secondary structural elements in BLC were summarized in Table 3. According to the data of Table 3, tRES had no significant effect on the secondary structure of BLC.

**Table 3:** The percentage of secondary structure elements of BLC in the presence and absence of tRES at 298 K (25 °C).

**[tRES] μM**	**Secondary structure content in BLC (%)**			
	**α-helix**	**β-sheet**	**β-turns**	**random coil**
0	27.4	21.2	17.6	33.8
10	27.6	21	17	33.3
20	28.3	20.4	18.8	33.6

### Analysis of binding mode and thermodynamic

For the static quenching mechanism, the number of binding sites (*n*) and binding constant (*K*) can be calculated by the following equation (8):

(8) $$\log\frac{F_{0}-F}{F}=\log K+n\log[Q]$$

**Figure 8:**
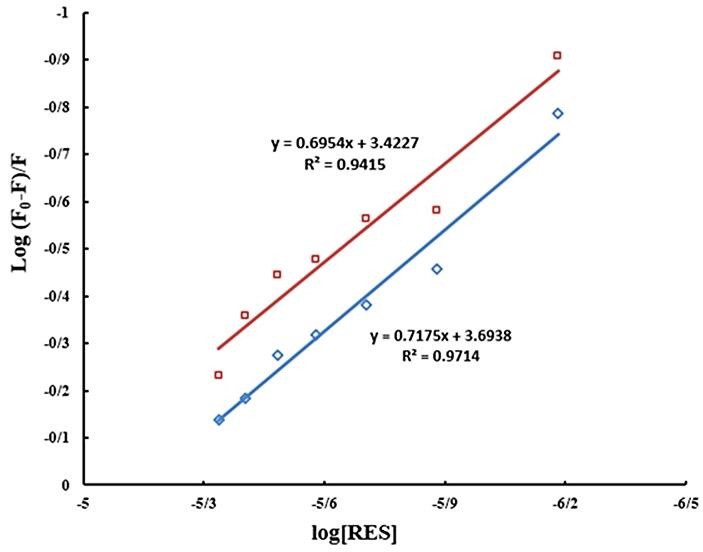
The linear plot of logF_0_-F/F vs. log [Q] for quenching of BLC in the presence of tRES at 298K (♦) and 310K (■).

The "*n*" and *K* values were determined by using the intercept and slope values of the plot logF_0_-F/F *vs*. log [*Q*] (Figure 8) and were listed in Table 2. The results showed that "*n*" value is approximately close to one, describe that one tRES molecule can bind to one BLC molecule and exist one binding site on BLC for tRES. The *K* values reduced by an increase in the temperature indicate that the stability of BLC and tRES complex lost. Also, *K* is of the order of 10^3^, indicates that there is a significant interaction between tRES and BLC.^30^

The interaction forces between a ligand and a macromolecule mainly including: electrostatic interactions, hydrophobic interactions, van der Waals forces and hydrogen bonding. The type of acting forces is estimated on the base of the sign and amount of ∆H and ∆S (thermodynamic parameters): ∆H˂ 0 and ∆S > 0 suggest electrostatic forces are dominant; ∆H ˃ 0 and ∆S ˃ 0 indicate hydrophobic interactions are main; ∆H ˂ 0 and ∆S ˂ 0 imply van der Waals forces and hydrogen bonding are more important.^19^ Equations from (9) to (11), were applied to calculating the ∆H (enthalpy), ∆G (Gibbs free energy) and ∆S (entropy) values, respectively, and the findings were summarized in Table 2.

(9) $$\ln\frac{K_{2}}{K_{1}}=\frac{-\Delta H}{R}(\frac{1}{T_{2}}-\frac{1}{T_{1}})$$

(10) ΔG=−RTlnK

(11) $$\Delta G^{}=\Delta H-T\Delta S$$

The negative values for ∆G, ∆H and ∆S support the assumption that tRES binds to BLC spontaneously, the formation complex between them (BLC-tRES) is exergonic reaction and entropy reduces during the formation of complex, respectively.^30^ Also, van der Waals forces and hydrogen bonds play main roles in this interaction.

### Molecular docking results

**Figure 9:**
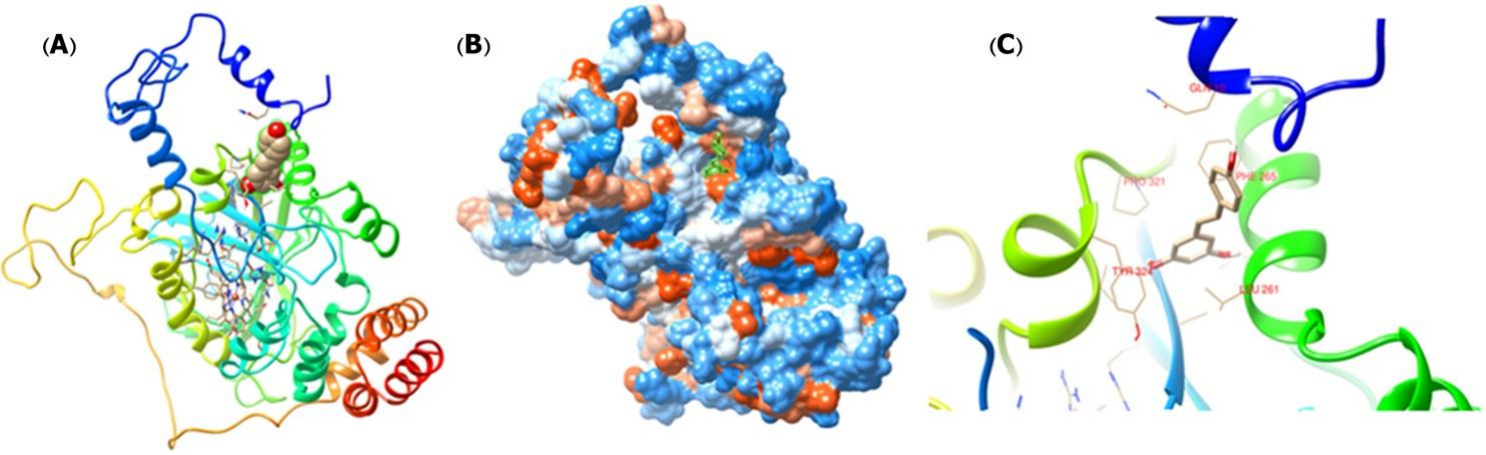
Molecular docking models for BLC-tRES complex (A) The structure of one subunit of BLC binding to tRES in carton form and (B) in hydrophobic surface model. (C) Detailed illustration of the binding between BLC and tRES.

Molecular docking is one of the most important methods which predicts the best interaction between a ligand and macromolecule based on the lowest energy.^37^ The molecular docking results illustrated that there are one binding site for tRES on BLC at a cavity among the β-barrel and helical domain which is away from heme group (Figure 9A and 9B). Also Figure 9C shows that in this interaction Phe 265 and Tyr 324 residues are involved. These results are in good agreement with results of UV absorption and fluorescence studies. Docking results indicate that hydrogen bonds and van der Waals forces contribute in interactions between BLC and tRES. The value of binding energy was obtained -6.8 Kcal mol^-1^ by docking calculation.

## Conclusion

The present work examined the effect of tRES on catalase structure and catalytic activity. *In vitro* studies indicated that, tRES can inhibit catalase activity by an uncompetitive mechanism and it spontaneously binds to BLC through one binding site. Spectroscopic results showed that tRES causes the conformational changes in BLC structure. Synchronous fluorescence, CD and UV-vis absorption spectroscopic studies suggested that the micro- region of Trp, Tyr and secondary structure of catalase change in the presence of tRES.

Fluorescence spectroscopy results suggest that with additional concentration of tRES, Trp residues are exposed to a less hydrophobic environment, due to unfolding of BLC structure upon interaction with tRES. Molecular docking calculation was applied in order to estimate the best mode of interaction between tRES and BLC, the binding energy of the interaction and the regions involved in the interaction, which the results show good agreements with the experimental binding studies data. Our results from thermodynamic studies indicated that tRES interacts with BLC through non-covalent and caused inhibition of the BLC activity.

## Ethical Issues

Not applicable.

## Conflict of Interest

The authors declare no conflict of interests.
